# SDG5 Gender Equality during the COVID-19 pandemic: an international comparative policy assessment: Ellen Kuhlmann

**DOI:** 10.1093/eurpub/ckac131.103

**Published:** 2022-10-25

**Authors:** E Kuhlmann, G Lotta, M Fernandez, A Herten-Crabb, J-M Maple, L MacFehr, L Paina, C Wenham, K Willis

**Affiliations:** 1 Clinic for Rheumatology and Immunology, Medizinische Hochschule Hannover, Hannover, Germany; 2 Department of Public Administration, Getulio Vargas Foundation, Sao Paulo, Brazil; 3 Universidade de Brasília, Brasilia, Brazil; 4 London School of Economics, London, UK; 5 University of Victoria, Melbourne, Australia; 6 Johns Hopkins Bloomberg School of Public Heal, Boston, USA

## Abstract

**Background:**

The COVID-19 pandemic caused severe disruptions in healthcare systems and societies and exacerbated existing inequalities for women and girls across every sphere. Our study explores health systems responses to gender equality goals during the COVID-19 pandemic and which role these goals play in pandemic recovery policies.

**Methods:**

We apply a qualitative comparative approach. Country case studies (expert information, secondary sources) were collected in March/April 2022. The sample comprised Australia, Brazil, Germany, United Kingdom and USA, reflecting conditions of high to upper-middle income countries with established public health systems, democratic political institutions and gender equality policies. Selected topics: maternity care/reproductive services, violence against women, and gender equality/female leadership.

**Results:**

All countries tried to keep essential maternity and reproductive services open, but strong limitations applied especially for prevention and counselling services; at the same time, digitalisation/telemedicine supported service expansion. Violence against women and children strongly increased during the pandemic. Routine services were partly kept open and new helplines occasionally established, but no action was taken to scale-up mental health support and respond to new demand. A push-back of gender equality was observed across countries in all areas of health and social care, often coupled with strong increase in intersecting social inequalities; participation of women in decision-making bodies was generally weak and not monitored.

**Conclusions:**

Across countries, gender equality policies cracked under the pressure of the COVID-19 pandemic; this is true for countries with male and female political leaders, and for different areas of SDG5 and health. There is an urgent need for more effective intersectional gender equality policies and improved participation of women in global health and in health system recovery plans.

**Key messages:**

• Health systems failed to take action to protect SDG5 goals; gender and intersecting inequalities strongly increased during the pandemic.

• Building back better after COVID-19 will only be possible with an intersectional gender equality programme and feminist policy approaches.

